# Agreement between large language models, human reviewers, and authors in evaluating STROBE checklists for observational studies in rheumatology

**DOI:** 10.1016/j.ero.2026.100193

**Published:** 2026-05-28

**Authors:** Emre Bilgin, Ebru Öztürk, Meera Shah, Lisa Traboco, Rebecca Everitt, Ai Lyn Tan, Marwan Bukhari, Vincenzo Venerito, Latika Gupta

**Affiliations:** 1 Department of Internal Medicine, Division of Rheumatology, Faculty of Medicine, Sakarya University, Sakarya, Turkiye; 2 Department of Biostatistics, Faculty of Medicine, Hacettepe University, Ankara, Turkiye; 3 Department of Rheumatology, Topiwala National Medical College and B.Y.L. Nair Hospital, Mumbai, India; 4 St Luke's Medical Center (Global City), Section of Rheumatology, Department of Medicine, Taguig, Philippines; 5 University of the Philippines, Medical Informatics Unit, Manila, Philippines; 6 New Cross Hospital, The Royal Wolverhampton Trust, Wolverhampton, UK; 7 NIHR Leeds Biomedical Research Centre, Leeds Teaching Hospitals NHS Trust, Leeds, UK; 8 Leeds Institute of Rheumatic and Musculoskeletal Medicine, University of Leeds, Leeds, UK; 9 Lancaster University Medical School, Lancaster University, Lancaster, UK; 10 Department of Rheumatology, Royal Lancaster Infirmary, Lancaster, UK; 11 Rheumatology Unit, Department of Precision and Regenerative Medicine and Ionian Area (DiMePReJ), University of Bari “Aldo Moro”, Bari, Italy; 12 Department of Rheumatology, Royal Wolverhampton Hospitals NHS Trust, Wolverhampton, UK; 13 School of Infection, Inflammation and Immunology, College of Medicine and Health, University of Birmingham, Birmingham, UK; 14 Francis Crick Institute, Camden, London, UK

## Abstract

**Objectives:**

Evaluating compliance with the Strengthening the Reporting of Observational Studies in Epidemiology (STROBE) statement can be time-consuming and subjective. This study compares STROBE assessments from large language models (LLMs), a human reviewer panel, and the original manuscript authors in observational rheumatology research.

**Methods:**

Guided by the Guidelines for Reporting Reliability and Agreement Studies and Development, Evaluation, and Assessment of Large Language Model Pathway B frameworks, 17 rheumatology articles (11 cohort, 4 cross-sectional, and 2 case-control) were independently assessed. Evaluations used the 22-item STROBE checklist, completed by the authors, a 5-person human panel (ranging from junior to senior professionals), and 2 LLMs (ChatGPT-5.2 and Gemini 3 Pro). Interrater reliability was calculated using Gwet’s Agreement Coefficient (AC1) with 95% CIs.

**Results:**

Overall agreement across all reviewers was 85.0% (AC1 = 0.826 [95% CI: 0.801-0.851]). Domain stratification showed almost perfect agreement for ‘Presentation & Context’ (AC1 = 0.841 [95% CI: 0.810-0.872]) and substantial agreement for ‘Methodological Rigor’ (AC1 = 0.803 [95% CI: 0.761-0.845]). Although LLMs achieved complete agreement with all human reviewers on standard formatting elements, their agreement declined on complex methodological items, with some pairwise comparisons yielding negative AC1 values. Intra-LLM and cross-version agreement across repeated independent runs was high, and estimates were stable across publication periods, providing no clear evidence of data leakage.

**Conclusions:**

While LLMs show potential for basic STROBE screening, their lower agreement with human experts on complex methodological items likely reflects a reliance on surface-level information. These models appear more reliable for standardising straightforward checks than for replacing expert human judgement in evaluating observational research.

**WHAT IS ALREADY KNOWN ON THIS TOPIC**

• Evaluating adherence to the STROBE statement is often subjective and time-consuming because the checklist lacks strictly objective criteria.
• Although large language models (LLMs) are increasingly used for various academic tasks, their reliability in evaluating complex methodological reporting remains poorly established.

**WHAT THIS STUDY ADDS**

• LLMs showed near-complete agreement with human reviewers and manuscript authors in identifying standard structural components and basic formatting elements of the STROBE checklist.
• On complex methodological items, LLM evaluations diverged from both human reviewers and authors; additionally, the models exhibited performance variations and varying levels of agreement among themselves.
• Intra-LLM and cross-version agreement across repeated independent runs was consistently high, suggesting that single-pass evaluations are relatively stable under a structured prompting protocol, while also confirming that some degree of within-model variability remains inherent to these systems.

**HOW THIS STUDY MIGHT AFFECT RESEARCH, PRACTICE OR POLICY**

• Language models can be utilised as supplementary tools to assist in the initial screening and standardisation of straightforward reporting checks.
• Despite their utility for screening straightforward reporting elements, expert human judgement remains necessary for evaluating complex methodological items in observational research.

Alt-text: Unlabelled box dummy alt text

## INTRODUCTION

Since the introduction of earlier models such as Generative Pre-trained Transformer 3, large language models (LLMs) have quickly become a part of academic research [1]. They are increasingly used for tasks such as literature screening, data extraction, and code generation, helping to reduce manual workload and human error [2]. However, their use in critical scientific processes is still debated, mainly because of unresolved issues related to reliability, bias, and transparency [3].

Transparent reporting is crucial for assessing observational research. The Strengthening the Reporting of Observational Studies in Epidemiology (STROBE) Statement provides a 22-item checklist to standardise this process and improve interpretability [4]. In practice, however, evaluating STROBE adherence is time-consuming and subject to interrater variability because it lacks strictly objective criteria [5]. Furthermore, as Sharp et al [5] noted, many authors view the checklist as a time-consuming administrative requirement rather than a practical tool, which can compromise the accuracy of self-reported adherence.

In this study, we aimed to systematically evaluate how well current LLMs can assess adherence to the STROBE checklist in observational rheumatology studies. By directly comparing their performance with a panel of human reviewers who have different levels of clinical expertise, as well as with the original manuscript authors, we sought to explore the potential of artificial intelligence (AI) to serve as a supplementary tool for standardising reporting assessments and helping readers and researchers in the critical appraisal of observational literature.

## METHODS

The study design, interrater agreement analyses, and reporting framework were guided by the principles of the Guidelines for Reporting Reliability and Agreement Studies and aligned with the Development, Evaluation, and Assessment of Large Language Models (DEAL) Pathway B checklist for the applied evaluation of pretrained models [6,7].

### Search strategy and selection criteria

A list of top rheumatology journals, ranked by Impact Factor, was compiled using the Observatory of International Research [8]. Journal-specific STROBE requirements were verified via prior bibliometric analyses and the respective ‘Guide for Authors’ guidelines [9,10]. Within the shortlisted journals, a structured search identified all articles that mentioned ‘STROBE’ published between February 2020 and February 2025. Seventeen articles containing complete, accessible STROBE checklists were included for evaluation; those comprised 11 cohort, 4 cross-sectional and 2 case-control studies (for full references, Supplementary Text 1). The selection process is detailed in the study selection flowchart (Supplementary Fig S1).

### Evaluation protocol

A panel of human reviewers, comprising 3 junior, 1 midlevel, and 1 senior professional, assessed the 17 articles independently using the STROBE checklist. Additionally, 2 LLMs, ChatGPT-5.2 and Gemini 3 Pro, conducted assessments. For comparison, the authors' original, self-reported STROBE checklists were also collected. All reviewers (human and machine) categorised each STROBE item as ‘Yes’ (adequately reported), ‘No’ (inadequately or not reported), or ‘Not applicable’. Each item and subitem was scored separately, resulting in a total of 34 individual judgements per article per reviewer.

To ensure reproducibility and adhere to the DEAL-B framework, evaluations used the premium subscription levels of 2 widely used, proprietary, closed-source models via their official web interfaces: ChatGPT-5.2 (Auto mode; OpenAI, accessed via chat.openai.com) and Gemini 3 Pro (Google LLC, accessed via gemini.google.com). All primary evaluations were performed on January 10, 2026. Web interfaces were chosen over application programming interface to reflect the real-world workflow of researchers, with generation parameters such as temperature and top-p maintained at vendor default settings. Each manuscript was assessed in a new, independent chat session to ensure independent evaluations. In each session, the following materials were provided to the models: the STROBE checklist (in PDF format) as the reference standard; 2 calibration articles with STROBE adherence scores (in Excel format), completed by an external evaluator not involved in the main study; and the target manuscript (in PDF format). We employed a 3-step prompting method. This included the following: (i) instructing the models to evaluate only reporting adherence rather than methodological quality, using strict Y/N/NA categories, and advising them not to infer missing details; (ii) a few-shot calibration step with 2 example articles with human reference scores to set the threshold for adequate reporting; and (iii) the final evaluation, which involved a simple 2-column table without explanations or reasoning to prevent the models from generating fabricated explanations. The full prompt texts and a complete list of the materials provided are available in Supplementary Text 2. All model outputs were recorded exactly as they were generated, without human modification.

### Statistical analysis

We assessed interrater reliability rather than diagnostic accuracy (eg, sensitivity), because evaluating reporting guidelines inherently relies on subjective interpretation and lacks an absolute 'ground truth'.

The primary analysis examined pairwise agreement between LLMs and human reviewers across all STROBE items and domains. Author comparisons, item-level analyses, model-to-model comparisons, sensitivity analyses, including intra-LLM and cross-version agreement, and agreement by publication period were treated as secondary or exploratory analyses. Before beginning data collection, our research team grouped STROBE items into 2 categories: 'Methodological Rigor' (Items 4-12) and 'Presentation & Context' (Items 1-3, 13-22). This matches the original STROBE checklist, in which items 4-12 constitute the 'Methods' section. We planned this grouping a priori to see if the models perform differently when evaluating core methodological steps and general reporting practices.

Interrater reliability across human reviewers, LLMs, and original manuscript authors was quantified using overall agreement proportions and Gwet’s First-Order Agreement Coefficient (AC1) with 95% CIs by using ‘irrCAC’ package [11]. Gwet's AC1 was selected to estimate reliability, as it corrects for chance agreement while avoiding the prevalence paradox frequently encountered with Cohen's kappa in highly skewed checklist datasets. Reliability estimates were interpreted using standard thresholds: <0.00 (poor), 0.00-0.20 (slight), 0.21-0.40 (fair), 0.41-0.60 (moderate), 0.61-0.80 (substantial), and 0.81-1.00 (almost perfect) [12]. All statistical analyses to evaluate overall, domain-specific, and item-specific agreement across all evaluator configurations were conducted using R software version 4.4.3 (R Foundation for Statistical Computing).

As a sensitivity analysis, intra-LLM and cross-version agreement were assessed in a subset of 9 articles selected to represent different study designs and publication periods. For ChatGPT-5.2, intra-LLM agreement was assessed via 2 additional independent runs performed on April 5, 2026, using the same model version and prompt protocol, for a total of 3 runs per article. For the Gemini runs, access to the original Gemini 3 Pro version was no longer available at the time of re-evaluation; Gemini 3.1 was therefore used for the repeated assessments to assess cross-version agreement as a proxy of intra-LLM agreement. To explore potential data leakage, overall agreement was also calculated separately for articles published in 2022 or earlier (n = 9) and in 2023 or later (n = 8).

## RESULTS

### Overall and domain-specific agreement

Of the 189 screened manuscripts, only 17 articles provided a complete and accessible STROBE checklist suitable for analysis (Supplementary Fig S1). Overall agreement across all items and reviewers (human, LLMs, authors) was 85.0% (AC1 = 0.826 [95% CI: 0.801-0.851]), indicating almost perfect agreement. Stratification by domain revealed almost perfect agreement for ‘Presentation & Context’ (AC1 = 0.841 [95% CI: 0.810-0.872]) and substantial agreement for ‘Methodological Rigor’ (AC1 = 0.803 [95% CI: 0.761-0.845]), with all *P* values < .001. Pairwise analysis demonstrated highly consistent interrater reliability among all evaluation groups, with the vast majority of combinations exhibiting almost perfect agreement (AC1 > 0.80; Figure 1, Figure 2A,B). At the article level, overall AC1 ranged from 0.624 (95% CI: 0.464-0.784) to 0.931 (95% CI: 0.877-0.985), with greater variance observed in the ‘Methodological Rigor’ domain (AC1 = 0.532 [95% CI: 0.240-0.824] to 0.921 [95% CI: 0.816-1.000]) compared with ‘Presentation & Context’ (AC1 = 0.685 [95% CI: 0.487-0.883] to 0.946 [95% CI: 0.862-1.000]).

**Figure 1:**
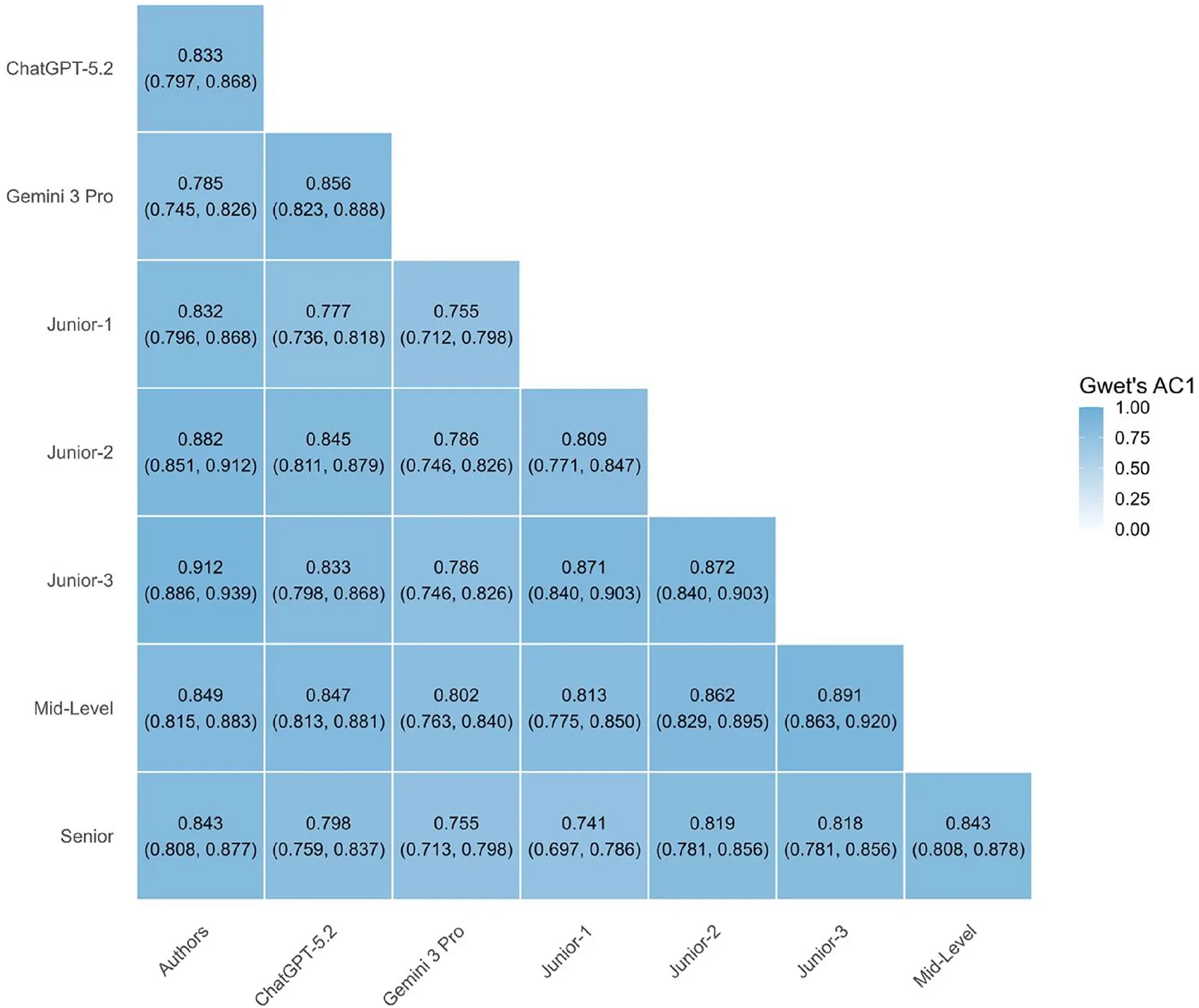
Overall pairwise agreement among reviewers. Heatmap displaying Gwet’s First-Order Agreement Coefficient (AC1) estimates with 95% CIs for overall pairwise comparisons across all STROBE checklist items. Reviewers include the original manuscript authors, 2 large language models (ChatGPT-5.2 and Gemini 3 Pro), and a panel of independent human reviewers (3 juniors, 1 midlevel, and 1 senior). The colour gradient indicates the strength of agreement, ranging from 0.00 (poor agreement; light blue) to 1.00 (perfect agreement; dark blue). STROBE, Strengthening the Reporting of Observational Studies in Epidemiology.

**Figure 2:**
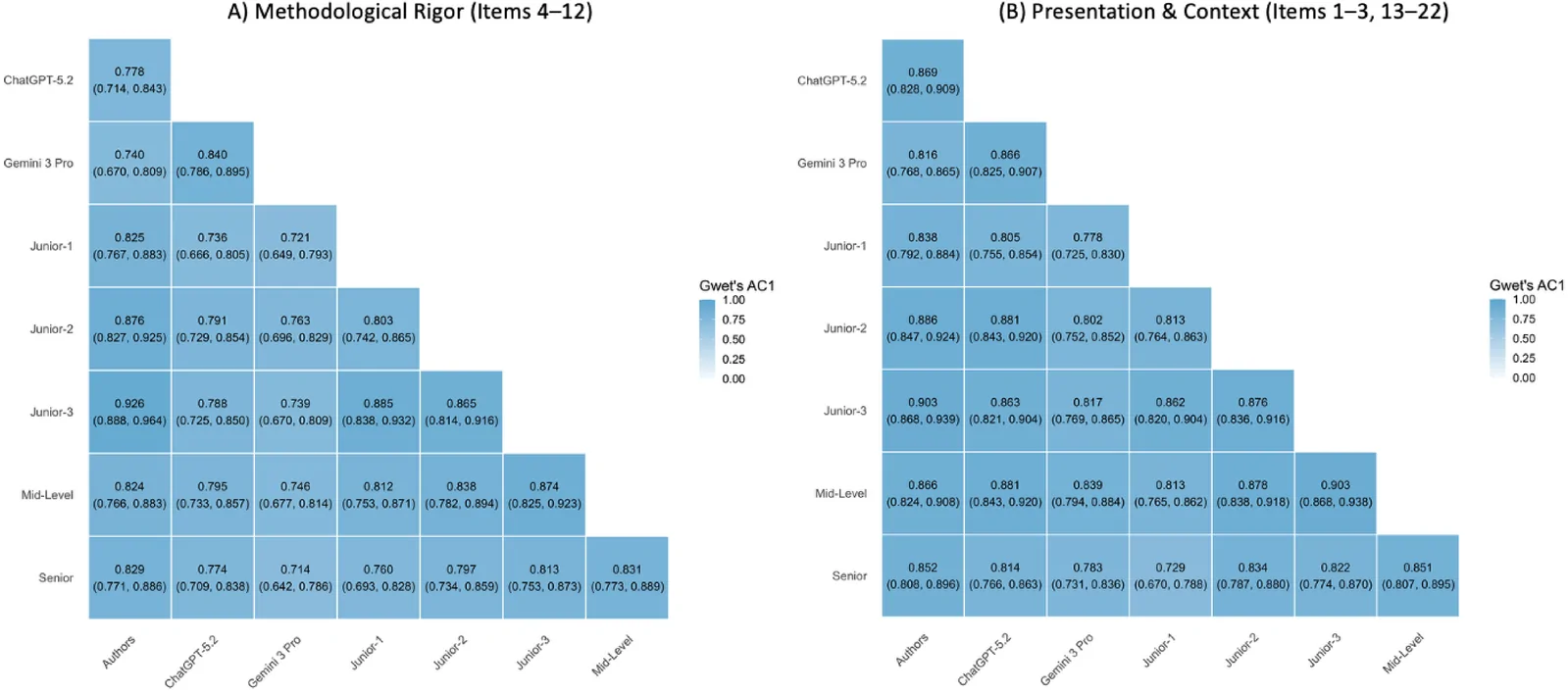
(A,B) Pairwise agreement stratified by STROBE domains. Heatmaps displaying Gwet’s First-Order Agreement Coefficient (AC1) estimates with 95% CIs for pairwise comparisons across 2 distinct evaluation domains: (A) Methodological Rigor (Items 4-12) and (B) Presentation and context (Items 1-3, 13-22). Reviewers include the original manuscript authors, 2 large language models (ChatGPT-5.2 and Gemini 3 Pro), and a panel of independent human reviewers (3 juniors, 1 midlevel, and 1 senior). The colour gradient indicates the strength of agreement, ranging from 0.00 (poor agreement; light blue) to 1.00 (perfect agreement; dark blue).

### Item-level analysis

An examination of the agreement scores for individual STROBE items and subitems revealed that while reviewers reached near-perfect agreement on many components, concordance naturally varied across the checklist. Noticeable differences, and in some cases, considerably lower scores, emerged on specific questions. Pairwise comparisons for each specific item across all evaluator groups are provided in Supplementary Figures S2 and S3. Pairwise CIs for individual item-level comparisons were not calculated, given the large number of evaluator combinations across 34 items and subitems; overall item-level agreement with 95% CIs is reported in Supplementary Table S1.

### High agreement on standard reporting items

Within the ‘Presentation & Context’ domain, both language models consistently identified standard manuscript sections (Supplementary Fig S2). For basic reporting elements, such as the informative abstract (Item 1b), background and rationale (Item 2), specific objectives (Item 3), summary of key results (Item 18), study limitations (Item 19), and overall interpretation (Item 20), ChatGPT-5.2 and Gemini 3 Pro showed perfect agreement (AC1 = 1.000) with all human reviewers and the original authors. Agreement was similarly high for identifying funding sources (Item 22) and participant numbers (Item 14a).

### Discrepancies in complex methodological items

Item-level analysis showed greater variation, especially in methodological reporting, specifically in Item 12 (Supplementary Fig S3). Agreement between the LLMs and the senior reviewer declined on more complex items. For example, regarding loss to follow-up (Item 12d), LLM-to-LLM agreement was moderate (AC1 = 0.447), and both models showed fair agreement with the authors and junior reviewers. However, their agreement with the senior reviewer dropped significantly; the agreement between Gemini 3 Pro and the senior reviewer fell within the negative range (AC1 = −0.252). For sensitivity analyses (Item 12e), LLMs agreed substantially with each other (AC1 = 0.652) and moderately with the senior reviewer but showed slight to fair agreement with the authors (AC1 = 0.071 for ChatGPT-5.2 and 0.272 for Gemini 3 Pro). Finally, regarding reporting continuous-variable categorisations (Item 16b), LLMs agreed moderately with one another (AC1 = 0.564), and human groups largely agreed with one another; however, agreement between LLMs and human reviewers fell into the fair and slight categories.

### Performance variations between language models

Agreement levels also differed between the 2 language models themselves. When assessing how missing data were handled (Item 12c), despite substantial LLM-to-LLM agreement (AC1 = 0.668), junior reviewers showed low concordance with both models, and Gemini 3 Pro generally exhibited lower agreement with human reviewers than ChatGPT-5.2. The authors maintained substantial agreement with junior reviewers on this item. Model performance varied regarding reporting the number of participants with missing data (Item 14b). ChatGPT-5.2 achieved substantial agreement with both authors (AC1 = 0.730) and the midlevel reviewer (AC1 = 0.722), whereas Gemini 3 Pro demonstrated fair (AC1 = 0.301) and moderate (AC1 = 0.450) agreement.

### Sensitivity analyses

As a sensitivity analysis, intra-LLM and cross-version agreement were assessed across 3 independent runs in a subset of 9 articles. For ChatGPT-5.2, overall intramodel agreement ranged from AC1 = 0.883 (95% CI: 0.842-0.925) to AC1 = 0.917 (95% CI: 0.882-0.952), with numerically higher agreement in the Presentation & Context domain (AC1 = 0.911 [95% CI: 0.865-0.958] to AC1 = 0.950 [95% CI: 0.915-0.985]) compared with Methodological Rigor (AC1 = 0.840 [95% CI: 0.764-0.917] to AC1 = 0.908 [95% CI: 0.848-0.967]). For Gemini, cross-version agreement between the original Gemini 3 Pro run and subsequent Gemini 3.1 runs ranged from AC1 = 0.836 (95% CI: 0.787-0.885) to AC1 = 0.915 (95% CI: 0.880-0.951), again with numerically higher agreement in the Presentation & Context domain (AC1 = 0.871 [95% CI: 0.814-0.927] to AC1 = 0.941 [95% CI: 0.903-0.980]) than in Methodological Rigor (AC1 = 0.784 [95% CI: 0.695-0.873] to AC1 = 0.875 [95% CI: 0.805-0.944]). Pairwise agreement heatmaps for both intra-LLM and cross-version analyses are provided in Supplementary Figure S4.

To explore potential data leakage, overall agreement was calculated separately for articles published in 2022 or earlier (n = 9) and those published in 2023 or later (n = 8). Agreement was similar across both groups: AC1 = 0.815 (95% CI: 0.777-0.853) for older articles and AC1 = 0.836 (95% CI: 0.8030.869) for more recent articles. Domain-level estimates were likewise comparable: for Methodological Rigor, AC1 = 0.787 (95% CI: 0.722-0.851) in the 2022-or-earlier group and AC1 = 0.817 (95% CI: 0.763-0.872) in the 2023-or-later group; for Presentation & Context, AC1 = 0.834 (95% CI: 0.787-0.880) and AC1 = 0.849 (95% CI: 0.808-0.890), respectively. CIs overlapped across all comparisons, providing no evidence that prior model exposure to older articles meaningfully inflated agreement estimates. Pairwise agreement heatmaps stratified by publication period are provided in Supplementary Figures S5 and S6.

## DISCUSSION

In this study, we examined the level of agreement between LLMs, a panel of human reviewers, and manuscript authors in assessing adherence to the STROBE checklist in observational rheumatology research. Although the overall agreement was 85.0%, this was primarily driven by the ‘Presentation & Context’ domain. Items in this section generally require identifying standard structural components, which aligns well with the basic text-matching nature of current language models [13]. In contrast, agreement was lower and more variable in the ‘Methodological Rigor’ domain, suggesting that evaluating complex methodological reporting remains challenging for all types of reviewers.

The item-level results suggest that a reviewer’s background likely influences their scoring patterns. Regarding the reporting of sensitivity analyses (Item 12e), authors generally agreed with junior reviewers but showed only slight to fair agreement with the LLMs. The models' scores were more aligned with those of the senior reviewer. This finding is consistent with existing research indicating that authors tend to view their own methodological reports more positively than independent reviewers do [14]. In our sample, junior reviewers appeared more inclined to accept authors’ statements, whereas senior experts and language models tended to apply stricter standards. Consequently, these findings suggest that incorporating LLMs might offer a useful supplementary check for verifying author-reported checklists. However, it should be acknowledged that the models' apparent strictness may not always reflect accurate judgement. The prompting protocol, specifically the instruction not to infer information that was not explicitly stated, likely contributed to conservative scoring on items such as loss to follow-up (Item 12d). Whether this conservatism reflects appropriate adherence to reporting standards or overcritical scoring of items that were, in fact, adequately reported cannot be determined without a ground-truth reference.

We also observed instances in which model assessments diverged from human reviewer judgements. For tasks such as handling loss to follow-up (Item 12d) and categorising continuous variables (Item 16b), the agreement between the models and the senior reviewer fell to slight or poor levels. This likely happens because the models rely heavily on literal wording. The negative Gwet’s AC1 values observed in certain pairwise comparisons for Items 12d and 12e reflect systematic disagreement beyond chance, most likely driven by fundamentally different interpretive standards between specific evaluator pairs on these items, rather than random scoring variability. If a paper does not clearly specify how loss to follow-up was managed, the model tends to consider it as missing data. In contrast, an experienced human reviewer can often infer this information from data tables or flowcharts. This is a recognised limitation: models generally perform well with surface-level text but struggle to recognise implicit methodological details [15]. Future studies should include qualitative analyses of these discordant cases to better understand the specific reasons behind these disagreements.

Apart from differences between human and model outputs, performance gaps were observed between the 2 language models. For example, in assessing missing data counts (Item 14b), ChatGPT-5.2 closely aligned with human reviewers, while Gemini 3 Pro had lower agreement scores. Prior research on medical benchmarks similarly indicates that outputs can vary with models' training and update cycles [16]. This highlights the importance of researchers carefully choosing their models, as the selected tool can significantly impact evaluation results.

Our results suggest that LLMs can contribute meaningfully to STROBE evaluations, although their usefulness varies considerably across checklist items. For items that require little more than confirming whether a standard section is present, such as the abstract, objectives, or funding statement, both models showed agreement with human reviewers that was comparable to interhuman agreement. In this subset of the checklist, LLM-assisted screening appears feasible. For methodological items, however, the findings are more cautious. When reviewers had to judge how loss to follow-up was handled or whether continuous variables were appropriately categorised, model assessments diverged from those of experienced raters more often than human reviewers diverged from each other. This pattern suggests that models respond to wording rather than to the underlying methodological intent, which limits their reliability for the more demanding parts of the checklist.

Our study has several key strengths. First, we assembled a diverse panel of human reviewers, including both junior researchers and senior experts, alongside the original manuscript authors. This setup enabled us to directly observe how clinical experience influences checklist scoring. Second, we tested 2 language models using a standardised, isolated-prompting protocol without human intervention, ensuring that our machine evaluations are reproducible. Third, we used Gwet’s AC1 statistic to measure agreement, which avoids the prevalence paradox often seen with Cohen’s kappa in checklist data, yielding a more accurate estimate of interrater reliability. However, our study also has limitations. The primary limitation is the small sample size of 17 articles, which naturally reduces statistical power and widens CIs, particularly for item-level analyses. However, this number was not a methodological choice but rather a direct reflection of the current literature. Despite screening a large number of observational studies in rheumatology, we found only 17 that explicitly reported using the STROBE checklist. While this small sample makes our estimates less stable, it also points to a broader, critical issue: the widespread lack of reporting of this guideline in our field. Since our sample consisted of articles from high-impact journals that generally mandate STROBE checklists, the reporting quality we evaluated is likely higher than average. This enrichment for well-reported studies may have inflated overall agreement estimates, and the findings may not generalise to observational research published in journals with less stringent reporting requirements, where LLM performance relative to human reviewers could differ considerably. Analytically, grouping the STROBE checklist into 2 domains helped us separate methods from formatting. We recognise that this split is imperfect; for example, Item 16b falls within the presentation domain, but its low agreement scores indicate it behaves more like a complex methodological task. We also evaluated only 2 commercial LLMs, excluding open-source models such as Llama and Mistral. We made this choice to reflect the daily reality of researchers, who generally rely on standard, ready-to-use web interfaces rather than on local installations that require specific hardware. Future studies should explore how open-source or specialised medical models perform in these tasks. In this study, each article was assessed once per model in the primary evaluation, mirroring the real-world workflow of human reviewers who typically read and score a manuscript just once. The sensitivity analysis showed that ChatGPT-5.2 produced highly consistent outputs across independent runs, and cross-version agreement for Gemini was similarly high, suggesting that single-pass evaluations are relatively stable under a structured prompting protocol. It should be noted that, as the original Gemini 3 Pro version was no longer accessible at the time of re-evaluation, Gemini 3.1 was used as a proxy model for the repeated runs; these results therefore reflect cross-version agreement rather than strict intramodel reliability. Some degree of within-model variability nonetheless remains, and future studies should consider systematic multirun designs, particularly for complex methodological items in which outputs may fluctuate more. Retrieval-augmented generation approaches, which anchor model outputs to specifically retrieved text segments, may offer a practical avenue for improving consistency in future applications [17]. Furthermore, the absence of a gold standard remains an inherent limitation of the current design. Future studies could address this by convening a panel of experienced methodologists to develop a consensus-based reference standard for STROBE adherence, against which LLM performance could then be evaluated in a formal accuracy study. Such an approach would allow a meaningful transition from reliability to accuracy research in this field. Another limitation is that STROBE items are nested within articles, which may introduce intra-article correlation. Treating items as independent observations may result in CIs that are somewhat narrower than those obtained with clustering-adjusted methods. With only 17 articles in our sample, cluster-robust approaches would themselves have limited reliability and were therefore not pursued; readers should keep this in mind when interpreting the precision of our estimates. There is also an inherent risk of data leakage. Because the evaluated articles are already public, they are likely part of the models’ training data. The models might have relied on prior exposure rather than extracting information solely from the texts we provided, which could skew the agreement rates upwards. Even with strict prompting to use only the provided text, it is impossible to separate an LLM from its pretraining memory. To assess this risk empirically, we compared overall agreement between articles published in 2022 or earlier and those published in 2023 or later; the absence of a meaningful difference between the 2 groups suggests that data leakage did not substantially inflate our estimates, although this analysis cannot fully exclude the possibility. Beyond this pretraining memory, the models themselves are constantly evolving; because we tested ChatGPT-5.2 and Gemini 3 Pro at a specific point in time, future updates may naturally yield different results.

In conclusion, language models show potential as practical tools for assessing general reporting standards, making them useful for basic STROBE screening. However, their agreement with senior human experts declines for complex methodological items, likely reflecting a reliance on surface-level information rather than deeper methodological understanding. While these models can help standardise straightforward checklist tasks, expert human judgement should remain essential for accurately evaluating study methods in observational research.

## CRediT authorship contribution statement

**Emre Bilgin:** Writing – review & editing, Writing – original draft, Project administration, Methodology, Investigation, Formal analysis, Data curation, Conceptualization. **Ebru Öztürk:** Methodology, Formal analysis. **Meera Shah:** Writing – review & editing, Investigation, Data curation, Conceptualization. **Lisa Traboco:** Writing – review & editing, Data curation, Conceptualization. **Rebecca Everitt:** Writing – review & editing, Data curation, Conceptualization. **Ai Lyn Tan:** Writing – review & editing, Methodology, Data curation, Conceptualization. **Marwan Bukhari:** Writing – review & editing, Data curation. **Vincenzo Venerito:** Writing – review & editing, Writing – original draft, Software, Methodology, Formal analysis, Data curation, Conceptualization. **Latika Gupta:** Writing – review & editing, Writing – original draft, Methodology, Formal analysis, Data curation, Conceptualization.

## Competing interests

All authors declare they have no competing interests.

## Appendix Supplementary materials

Image, application 1

## Footnotes

Supplementary material associated with this article can be found in the online version at doi:10.1016/j.ero.2026.100193.
