# Supplementary material for: Agreement between large language models, human reviewers, and authors in evaluating STROBE checklists for observational studies in rheumatology
Source: EULAR Rheumatol Open. 2026 May 28;2(3):100193. doi: 10.1016/j.ero.2026.100193 (PMC13650333; doi:10.1016/j.ero.2026.100193)
Supplement: Supplementary file 1 [file mmc1.docx]

**Supplementary Text-1:**

**List of Included Articles.** Complete references for the 17 observational studies assessed for the STROBE checklist agreement

1.	Larid, G., et al., *Detection of hypophosphatasia in hospitalised adults in rheumatology and internal medicine departments: a multicentre study over 10 years.* RMD Open, 2024. **10**(2).

2.	Bastard, L., et al., *Risk of serious infection associated with different classes of targeted therapies used in psoriatic arthritis: a nationwide cohort study from the French Health Insurance Database (SNDS).* RMD Open, 2024. **10**(1).

3.	Sloan, M., et al., *Prevalence and identification of neuropsychiatric symptoms in systemic autoimmune rheumatic diseases: an international mixed methods study.* Rheumatology (Oxford), 2024. **63**(5): p. 1259-1272.

4.	Crossfield, S.S.R., et al., *Changes in the pharmacological management of rheumatoid arthritis over two decades.* Rheumatology (Oxford), 2021. **60**(9): p. 4141-4151.

5.	Spierings, J., et al., *How do patients with systemic sclerosis experience currently provided healthcare and how should we measure its quality?* Rheumatology (Oxford), 2020. **59**(6): p. 1226-1232.

6.	Garriga, C., et al., *Clinical and molecular associations with outcomes at 2 years after acute knee injury: a longitudinal study in the Knee Injury Cohort at the Kennedy (KICK).* Lancet Rheumatol, 2021. **3**(9): p. e648-e658.

7.	Jones, C.A., et al., *The effect of geographic location and payor type on provincial-wide delivery of the GLA:D program for hip and knee osteoarthritis in Alberta, Canada.* Osteoarthr Cartil Open, 2023. **5**(4): p. 100398.

8.	Mehta, P., et al., *Gastrointestinal manifestations in systemic lupus erythematosus: data from an Indian multi-institutional inception (INSPIRE) cohort.* Rheumatology (Oxford), 2025. **64**(1): p. 156-164.

9.	Mertens, M.G., et al., *Prediction models for treatment success after an interdisciplinary multimodal pain treatment program.* Semin Arthritis Rheum, 2025. **70**: p. 152592.

10.	Palmowski, A., et al., *Initiation of glucocorticoids before entering rheumatology care associates with long-term glucocorticoid use in older adults with early rheumatoid arthritis: A joint analysis of Medicare and the Rheumatology Informatics System for Effectiveness (RISE) data.* Semin Arthritis Rheum, 2024. **68**: p. 152535.

11.	van Straalen, J.W., et al., *Methotrexate therapy associated with a reduced rate of new-onset uveitis in patients with biological-naïve juvenile idiopathic arthritis.* RMD Open, 2023. **9**(2).

12.	Li, H., et al., *Risk of gout flares after COVID-19 vaccination: A case-crossover study.* Semin Arthritis Rheum, 2022. **56**: p. 152059.

13.	Rempenault, C., et al., *Risk of diverticulitis and gastrointestinal perforation in rheumatoid arthritis treated with tocilizumab compared to rituximab or abatacept.* Rheumatology (Oxford), 2022. **61**(3): p. 953-962.

14.	Crossfield, S.S.R., et al., *Changes in ankylosing spondylitis incidence, prevalence and time to diagnosis over two decades.* RMD Open, 2021. **7**(3).

15.	Chua, J., et al., *Stakeholders' preferences for osteoarthritis interventions in health services: A cross-sectional study using multi-criteria decision analysis.* Osteoarthr Cartil Open, 2020. **2**(4): p. 100110.

16.	Van der Elst, K., et al., *One in five patients with rapidly and persistently controlled early rheumatoid arthritis report poor well-being after 1 year of treatment.* RMD Open, 2020. **6**(1).

17.	Roth, E., et al., *Gastroesophageal reflux disease is associated with a more severe interstitial lung disease in systemic sclerosis in the EUSTAR cohort.* Rheumatology (Oxford), 2025. **64**(Si): p. Si63-si72.

**Supplementary Text-2:**

Standardized Prompting Protocol. This text details the complete, three-stage prompt sequence (including constraints, few-shot calibration examples, and target evaluation structure) administered to the language models to assess STROBE reporting adherence.

***---------------------------------------------------------------------------------------------***

***#PROMPT-0 — Role, borders and logic of scoring***

You are acting as an independent rater assessing adherence to the STROBE (Strengthening the Reporting of Observational Studies in Epidemiology) checklist.

Your task is to evaluate whether each STROBE item is reported in a manuscript, based strictly on the content of the provided PDF files.

Scoring options:

- Y (Yes): the item is clearly and explicitly reported

- N (No): the item is not reported or only partially reported

- NA (Not applicable): the item is inherently not applicable to the study design

Important rules:

- This assessment concerns reporting adherence only.

- Do NOT evaluate methodological quality, correctness, or risk of bias.

- If information is incomplete, implicit, vague, or only partially reported, it must be scored as "N".

- Use "NA" only when the item is genuinely not applicable, not when information is missing.

- Do not infer or assume information that is not explicitly stated in the manuscript.

***#PROMPT-1- Calibration (2 articles and external responses)***

You are provided with the following files:

- "STROBE.pdf": the current STROBE checklist to be used as the reference standard.

- "article1.pdf" and "article2.pdf": two example observational studies.

- "training_scores.xlsx": STROBE adherence scores for article1 and article2, completed by an external human evaluator who is not part of the main study raters.

These examples are provided solely to illustrate how the scoring rules were applied in practice and the level of explicitness required for assigning a "Yes" score.

They are NOT provided as gold-standard answers, and they must NOT be used for training, optimization, or learning beyond calibration of reporting thresholds.

Please review the STROBE checklist and examine how the scoring rules were applied to article1 and article2.

Do not generate any new scores at this stage.

Acknowledge once you are ready to apply the same scoring rules consistently to a new manuscript.

**#PROMPT-2 - Article Scoring**

Now evaluate the attached manuscript using the same STROBE checklist and the same scoring rules applied previously.

Use the STROBE checklist provided in "STROBE.pdf".

Output requirements:

- Provide a table with exactly two columns:

1) STROBE item (including sub-items such as 1a, 1b)

2) Score (Y / N / NA)

Do not include explanations, justifications, page numbers, or comments.

Do not summarize or interpret the results.

Do not compare this manuscript to previous examples.

Produce a single, complete STROBE adherence table.

***---------------------------------------------------------------------------------------------***

**Supplementary Figure-1. Study selection flowchart.**

**Supplementary Figure 2**. **Item-specific pairwise inter-rater agreement for Presentation & Context items.** Heatmaps display Gwet's AC1 agreement scores for each individual item and sub-item within the Presentation & Context domain (Items 1–3 and 13–22) of the STROBE checklist. Each matrix compares the categorical reporting assessments (Yes / No / Not Applicable) among two large language models (ChatGPT-5.2 and Gemini 3 Pro), five independent human reviewers of varying clinical experience (Junior-1, Junior-2, Junior-3, Mid-Level, and Senior), and the original study authors. The color gradient indicates the strength of agreement, ranging from 0.00 (poor agreement; light blue) to 1.00 (perfect agreement; dark blue). Negative AC1 values, which indicate agreement below chance level, are displayed in gray. Items where all raters reached complete agreement (AC1 = 1.000) show zero variance; confidence intervals could not be computed for these cells and are not displayed. Pairwise confidence intervals for individual item-level comparisons are not reported in this figure; overall item-level agreement with 95% confidence intervals for each STROBE item is available in Supplementary Table 1.

**Supplementary Figure 3. Item-specific pairwise inter-rater agreement for Methodological Rigor items.** Heatmaps display Gwet's AC1 agreement scores for each individual item and sub-item within the Methodological Rigor domain (Items 4–12) of the STROBE checklist. Each matrix compares the categorical reporting assessments (Yes / No / Not Applicable) among two large language models (ChatGPT-5.2 and Gemini 3 Pro), five independent human reviewers of varying clinical experience (Junior-1, Junior-2, Junior-3, Mid-Level, and Senior), and the original study authors. The color gradient indicates the strength of agreement, ranging from 0.00 (poor agreement; light blue) to 1.00 (perfect agreement; dark blue). Negative AC1 values, which indicate agreement below chance level, are displayed in gray. Items where all raters reached complete agreement (AC1 = 1.000) show zero variance; confidence intervals could not be computed for these cells and are not displayed. Pairwise confidence intervals for individual item-level comparisons are not reported in this figure; overall item-level agreement with 95% confidence intervals for each STROBE item is available in Supplementary Table 1.

**Supplementary Figure 4. Intra-LLM and cross-version agreement across repeated independent runs.** Heatmaps display Gwet's AC1 agreement scores with 95% confidence intervals for pairwise comparisons across three independent runs of each language model, calculated in a subset of nine articles selected to represent different study designs and publication periods. The upper panel shows intra-model agreement results for ChatGPT-5.2, for which all three runs used the same model version (1st run: January 10, 2026; 2nd and 3rd runs: April 5, 2026). The lower panel shows cross-version agreement for the Gemini model; as the original Gemini 3 Pro version was no longer accessible at the time of re-evaluation, the 2nd and 3rd runs were performed using Gemini 3.1, and these comparisons therefore reflect cross-version rather than strict intra-model reliability. Results are presented separately for the Methodological Rigor domain (Items 4–12), the Presentation & Context domain (Items 1–3, 13–22), and overall. The color gradient indicates the strength of agreement, ranging from 0.00 (poor agreement; light blue) to 1.00 (perfect agreement; dark blue).

**Supplementary Figure 5.** **Pairwise agreement among all reviewers for articles published in 2022 or earlier (n=9).** Heatmap displaying Gwet's First-Order Agreement Coefficient (AC1) estimates with 95% confidence intervals for pairwise comparisons across all STROBE checklist items in the subgroup of articles published in 2022 or earlier. Reviewers include the original manuscript authors, two large language models (ChatGPT-5.2 and Gemini 3 Pro), and a panel of independent human reviewers (Junior-1, Junior-2, Junior-3, Mid-Level, and Senior). This analysis was performed as an exploratory sensitivity analysis to assess the potential influence of data leakage on agreement estimates. The color gradient indicates the strength of agreement, ranging from 0.00 (poor agreement; light blue) to 1.00 (perfect agreement; dark blue).

**Supplementary Figure 6.** **Pairwise agreement among all reviewers for articles published in 2023 or later (n=8).** Heatmap displaying Gwet's First-Order Agreement Coefficient (AC1) estimates with 95% confidence intervals for pairwise comparisons across all STROBE checklist items in the subgroup of articles published in 2023 or later. Reviewers include the original manuscript authors, two large language models (ChatGPT-5.2 and Gemini 3 Pro), and a panel of independent human reviewers (Junior-1, Junior-2, Junior-3, Mid-Level, and Senior). This analysis was performed as an exploratory sensitivity analysis to assess the potential influence of data leakage on agreement estimates. The color gradient indicates the strength of agreement, ranging from 0.00 (poor agreement; light blue) to 1.00 (perfect agreement; dark blue).

**Supplementary Table 1.** Overall inter-rater agreement for each STROBE item and sub-item

| Item Numbers | Agreement | Domain | Gwets AC1 | 95% Confidence Interval of Gwets AC1 | p-value |
| --- | --- | --- | --- | --- | --- |
| 1(a) | 0.887 | **Presentation & Context** | 0.869 | (0.724 – 1.000) | <0.001 |
| 1(b) | 1.000 | **Presentation & Context** | 1.000 | (Not estimable) | NA |
| 2 | 1.000 | **Presentation & Context** | 1.000 | (Not estimable) | NA |
| 3 | 1.000 | **Presentation & Context** | 1.000 | (Not estimable) | NA |
| 4 | 1.000 | Methodological Rigor | 1.000 | (Not estimable) | NA |
| 5 | 0.985 | Methodological Rigor | 0.985 | (0.953 – 1.000) | <0.001 |
| 6(a) | 1.000 | Methodological Rigor | 1.000 | (Not estimable) | NA |
| 6(b) | 0.708 | Methodological Rigor | 0.622 | (0.448 - 0.796) | <0.001 |
| 7 | 0.958 | Methodological Rigor | 0.957 | (0.862 – 1.000) | <0.001 |
| 8 | 1.000 | Methodological Rigor | 1.000 | (Not estimable) | NA |
| 9 | 0.842 | Methodological Rigor | 0.804 | (0.630 - 0.978) | <0.001 |
| 10 | 0.756 | Methodological Rigor | 0.713 | (0.561 - 0.864) | <0.001 |
| 11 | 0.954 | Methodological Rigor | 0.952 | (0.876 – 1.000) | <0.001 |
| 12(a) | 1.000 | Methodological Rigor | 1.000 | (Not estimable) | NA |
| 12(b) | 0.767 | Methodological Rigor | 0.713 | (0.511 - 0.915) | <0.001 |
| 12(c) | 0.630 | Methodological Rigor | 0.499 | (0.263 - 0.735) | <0.001 |
| 12(d) | 0.517 | Methodological Rigor | 0.306 | (0.190 - 0.421) | <0.001 |
| 12(e) | 0.578 | Methodological Rigor | 0.399 | (0.194 - 0.605) | <0.001 |
| 13(a) | 0.943 | **Presentation & Context** | 0.941 | (0.842 – 1.000) | <0.001 |
| 13(b) | 0.660 | **Presentation & Context** | 0.561 | (0.328 - 0.793) | <0.001 |
| 13(c) | 0.729 | **Presentation & Context** | 0.675 | (0.468 - 0.881) | <0.001 |
| 14(a) | 0.956 | **Presentation & Context** | 0.954 | (0.899 – 1.000) | <0.001 |
| 14(b) | 0.588 | **Presentation & Context** | 0.450 | (0.280 - 0.621) | <0.001 |
| 14(c) | 0.620 | **Presentation & Context** | 0.490 | (0.278 - 0.701) | <0.001 |
| 15 | 0.891 | **Presentation & Context** | 0.884 | (0.792 - 0.977) | <0.001 |
| 16(a) | 0.905 | **Presentation & Context** | 0.901 | (0.810 - 0.991) | <0.001 |
| 16(b) | 0.613 | **Presentation & Context** | 0.456 | (0.249 - 0.663) | <0.001 |
| 16(c) | 0.637 | **Presentation & Context** | 0.530 | (0.331 - 0.728) | <0.001 |
| 17 | 0.821 | **Presentation & Context** | 0.799 | (0.632 - 0.966) | <0.001 |
| 18 | 1.000 | **Presentation & Context** | 1.000 | (Not estimable) | NA |
| 19 | 1.000 | **Presentation & Context** | 1.000 | (Not estimable) | NA |
| 20 | 1.000 | **Presentation & Context** | 1.000 | (Not estimable) | NA |
| 21 | 0.985 | **Presentation & Context** | 0.985 | (0.953 – 1.000) | <0.001 |
| 22 | 0.985 | **Presentation & Context** | 0.985 | (0.953 – 1.000) | <0.001 |

Agreement proportions and Gwet's First-Order Agreement Coefficient (AC1) with 95% confidence intervals are reported for each STROBE item and sub-item across all raters (authors, ChatGPT-5.2, Gemini 3 Pro, and five human reviewers). The p-values for AC1 reflect the probability that the observed agreement exceeds chance-level agreement. For items where all raters reached complete agreement (AC1 = 1.000), variance is zero. Therefore, for those AC1 estimates confidence intervals cannot be computed; these are reported as “Not estimable” and p-values are not defined (reported as NA).
